# Retinal contour modelling to reproduce two-dimensional peripheral spherical equivalent refraction

**DOI:** 10.1364/BOE.426413

**Published:** 2021-06-08

**Authors:** Qing Li, Fengzhou Fang

**Affiliations:** 1 Center of Micro/Nano Manufacturing Technology (MNMT-Dublin), University College Dublin, Dublin 4, Ireland; 2 State Key Laboratory of Precision Measuring Technology and Instruments, Laboratory of Micro/Nano Manufacturing Technology (MNMT), Tianjin University, Tianjin 300072, China

## Abstract

Reproduction of the peripheral spherical equivalent refraction (SER) in the eye model is critical for investigations in myopia control. Based on the derivation of a linear relationship between SER and the vergence of the wavefront at exit pupil center, a computing method is proposed to locate the retinal points to reproduce the two-dimensional (2D) distribution of SER. The method is validated by reproducing SER maps measured on both emmetropic and myopic eyes in a realistic eye model based on measurement data. By fitting the retinal points to a general ellipsoid, the limited capability of the general ellipsoid model in reproducing the 2D map of SER is calculated and compared with original data. The high accuracy in SER reproduction and low time-cost of the proposed retinal-locating method can help significantly improve the precision and accuracy of customized wide-angle eye modelling.

## 1. Introduction

Although it remains a question whether peripheral myopic defocus is an active factor in slowing myopic progression, evidence from various sources [1–4] have shown that the image quality experienced by the retina can affect the growth of the human eye. Specifically, it is believed that peripheral myopic defocus may help prevent myopic progression, which has been widely applied in corrective lenses designed for myopia control [5]. Moreover, findings from animal studies have shown that the retina can detect optical defocus locally and grow accordingly [6]. Therefore, reproduction of the peripheral refraction is critical in human eye modelling for applications in myopia control.

Accordingly, wide-angle eye models have been developed to reproduce the axial and peripheral aberrations measured in the human population. Specifically, the procedure of building customized eye models has been developed to reconstruct the optical features measured in the individual eye. However, most of the customized eye models [7–10] are built based on optimizing certain parameters of ocular components, which means the complexity of the eye model is limited by the efficiency of optimization. Therefore, the irregular patterns of peripheral defocus as measured in the human eye cannot be fully reproduced by optimizing a limited number of ocular parameters.

Understanding the relationship between the ocular structure with its optical properties is fundamental in human eye modelling. It may help derive certain structural properties directly from optical features without going through blind optimization. Most of the previous studies in this field focus on the optical impacts of the optical components such as refracting surfaces [11–13], pupil size [14] and orientation of the ocular axes [15]. However, the optical contributions of those components are interdependent, which makes it hard to isolate the exact source of optical features. On the contrary, the retinal surface topography does not affect the propagation of light beam through the eye, and thus is a distinct factor that determines the curvature of the initial wavefront entering the first refracting surface. By representing the retina as a conic surface, Dunne [16] studied the contribution of retinal curvature to the sagittal and tangential peripheral refractive errors, which was applied in generating peripheral refraction by adjusting both corneal and retinal surface geometry. Dunne’s method was further analysed by Verkicharla et al. [17], who found that there were significant differences in the retinal shapes obtained based on Dunne’s method compared with those measured by magnetic resonance imaging (MRI). Moreover, the method proposed by Dunne involves a manipulation of corneal topography, which can be easily measured with current technology and thus should not be adjusted in eye modelling.

Furthermore, the retinal shapes in most of the previous wide-angle optical eye models were simplified as a sphere or ellipsoid [8,10,18]. The investigation on the retinal contour was thus limited to the discussion of its curvature and asphericity. However, studies using magnetic resonance imaging have found that the real retinal contour is not regular [19], which may produce significant effects on peripheral defocus. Therefore, it remains a question whether quadratic surface modelling of retina is sufficient to reconstruct the irregular patterns of peripheral defocus measured in human eye.

In this study, a linear relationship between spherical equivalent refraction (SER) and the vergence of the wavefront at exit pupil center is established and verified. Based on this finding, a method of locating the retinal contour for a given eye model is proposed that can reproduce a specific two-dimensional distribution of SER typically measured in the human eye. It is believed that the proposed modelling method is a powerful tool for characterizing the irregularities of peripheral refraction distribution, which is instrumental in the design and evaluation of customized lenses for myopia control.

## 2. Theoretical approaches

### 2.1 Description of the retinal contour

In this paper, the retinal contour is discretely represented by a set of retinal points on a set of chief rays passing through the exit pupil center of the eye model. Each retinal point corresponds to a certain visual field location. In this way, the position of each retinal point is determined by the distance from the exit pupil center to the retinal point and its visual field location.

**Fig. 1.**
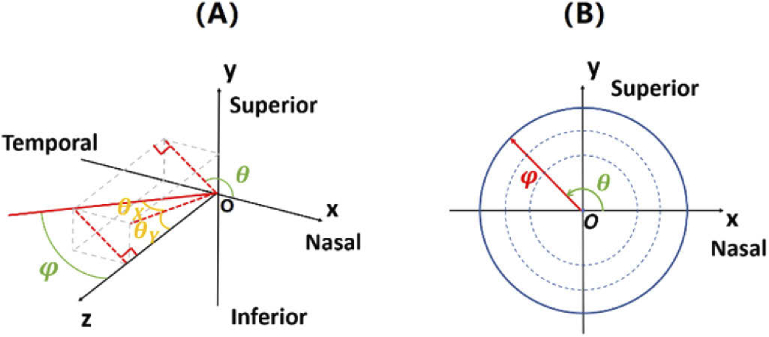
Representation of the visual field location. (A) Geometrical relationship between the polar (θ,φ) and Cartesian $\left(\theta_{X},\theta_{Y}\right)$ notations as shown for the right eye in the front view. Here X axis points towards the nose horizontally, Y axis points towards the forehead vertically, and Z axis is the primary line-of-sight pointing towards the object space. (B) The visual field position represented in the polar system in the front view of the eye. Each circle represents the field locations with the same visual field angle φ, while θ represents the meridian angle which ranges from 0 to 360 deg (360 deg not included). The units of all the angular parameters are degree.

There are two equivalent methods of representing the visual field location: (1) the polar method of visual field angle φ and meridian angle θ; and (2) the Cartesian method of horizontal $\theta_{X}$ and vertical angle $\theta_{Y}$. The two set of angles can be easily derived from each other from trigonometry, as shown in Fig. 1. If the positive value of $\theta_{X}$ and $\theta_{Y}$ represent temporal and inferior visual field, respectively, the transformations from Cartesian method to polar method can be derived by

(1.a) $$\varphi =\arccos(\cos\theta_{X}\cdot \cos\theta_{Y}),$$

(1.b) $$\theta =\left\{ \begin{array}{lc} \arccos(-\frac{\sin\theta_{X}}{\sin\varphi }), & \theta_{Y}<0\\ \text{arccos(}\frac{\sin\theta_{X}}{\sin\varphi }\text{)}+180\text{, } & \theta_{Y}>0\\ 0, & \theta_{Y}=0\text{, }\theta_{X}<0\\ \text{180, } & \theta_{Y}=0\text{, }\theta_{X}>0 \end{array}\right..$$

It should be mentioned that, when measuring the peripheral refractions based on head/eye rotation, the field location obtained by viewing superior targets correspond to the inferior visual field (or superior retina). Therefore, positive value of $\theta_{Y}$ is set for the inferior visual field for ease of application.

Since it is more suitable to reveal the patterns of peripheral optics, the polar method is applied throughout this paper for displaying the field maps of SER. However, as the Cartesian method is often used by experimental studies to report the measured peripheral optical properties, Eq. (1.a) and (1.b) can be utilized to transform the measurement data into the polar form.

### 2.2 Linear correlation theory

**Fig. 2.**
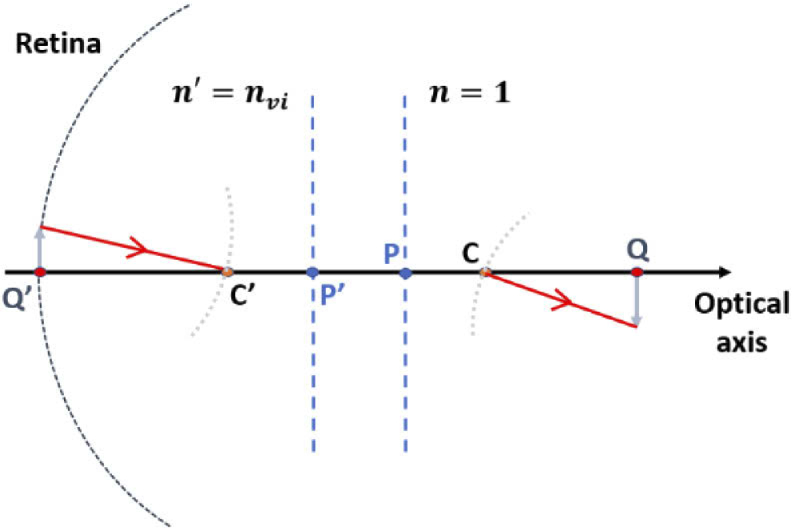
Diagram of paraxial optical imaging in ocular system. Q’, C’ and P’ respectively represent the axial position of the object point, exit pupil center, and principal point in the vitreous humor, while Q, C and P are their counterparts in the air.

Based on paraxial optical theory, the ocular optical system can be described in the diagram as shown in Fig. 2, where C’ and C are the exit and entrance pupil center of the eye, P’ and P the principal points in vitreous humor and air, Q’ and Q an arbitrary pair of conjugated object and image points, respectively.

Based on the Gaussian imaging equation, the imaging relationship between C’ and C can be described by

(2) $$\frac{n_{vi}}{l_{C^{\prime }}}+\Phi =\frac{1}{l_{C}},$$

where $l_{C^{\prime }}$ and $l_{C}$ are the distance from P’ to C’ and the distance from P to C, respectively; $n_{vi}$ is the refractive index of vitreous humor; Φ is the paraxial optical power. The distance parameters in this section follow the tradition that positive values correspond to the same direction as the light propagation. In this way, $l_{C^{\prime }}$ would be negative if C’ is to the left side of P’ in Fig. 2.

Similarly, for a pair of retinal point (Q’) and its image point in air (Q), we have

(3) $$\frac{n_{vi}}{l_{Q^{\prime }}}+\Phi =\frac{1}{l_{Q}}.$$

Assume $x_{Q^{\prime }}=l_{Q^{\prime }}-l_{C^{\prime }}$, $x_{Q}=l_{Q}-l_{C}$, then Eq. (3) can be transformed into

(4) $$\frac{n_{vi}}{x_{Q^{\prime }}+l_{C^{\prime }}}+\Phi =\frac{1}{x_{Q}+l_{C}}.$$

Combine Eq. (2) and (4), we have

(5) $$\frac{n_{vi}}{x_{Q^{\prime }}+\frac{n_{vi}}{\frac{1}{l_{C}}-\Phi }}+\Phi =\frac{1}{x_{Q}+l_{C}}.$$

Assume $V_{in}=n_{vi}/x_{Q^{\prime }}$, $V_{out}=1/x_{Q}$, Eq. (5) can be transformed into

(6) $$V_{out}={\left(\frac{1}{1-\Phi \cdot l_{C}}\right)}^{2}\cdot V_{in}+\frac{\Phi }{1-\Phi \cdot l_{C}}.$$

As can be seen, the vergence of the wavefront at entrance pupil center ($V_{out}$) is linear correlated to the wavefront at exit pupil center ($V_{in}$). In visual optics, $V_{out}$ can be considered as the opposite value of the refractive error of the eye.

In this paper, it is hypothesized that the linear correlation works for each visual field location in the ocular system. Namely, along each chief ray, the change in SER is approximately linear with respect to the change in the vergence of the wavefront at exit pupil center. In section 3.1, this pattern has been shown to work in both foveal and peripheral fields of a realistic eye model. And it can be applied to reproduce the SER map by determining the retinal points for all the visual field locations, as discussed in the next section.

### 2.3 Retinal-locating method for reproducing 2D map of SER

In this section, a procedure is presented to find the location of the retinal surface points required for the eye model to reproduce a specific (typically measured) two-dimensional distribution of SER. Here two-dimensional distribution (2D map) means the SER covers an area of the visual field rather than a certain meridian. It is assumed that all the structural parameters of the eye—except the retinal contour—have been set up previously. Given the direction of primary line-of-sight, the entrance and exit pupil centers and the coordinate system for reporting the wavefront aberration in central and peripheral visual field can be set up with respect to the global eye system. For the ease of presentation, the 2D map of SER as the target for modelling retinal contour is denoted by MAP0. It should be mentioned that both the 2D map of SER and the retinal contour in this study are composed of discrete data points, while interpolation can always be used to connect the data points into a surface. In this way, the retinal modelling procedure is performed for each field angle separately.

In total, three rounds of ray-tracing throughout the eye for the total visual field are needed to locate the retinal contour. The detailed steps are listed as follows.

• Step 1: Locating an initial set of retinal points (RP1)

Given MAP0, a series of spherical wavefronts with the vergence at the entrance pupil center set the same as MAP0 are traced into the eye along the chief rays corresponding to the field positions in MAP0. Then, the wavefronts emerged out of the exit pupil center in the vitreous humor are calculated and fit to Zernike polynomials up to the 6^th^ order, which can be used to derive the average radius of curvature:

(7) $$l_{{\text{C}}^{\prime }\text{P}1}=1/[\frac{1}{R^{2}}\cdot (4\sqrt{3}c_{2}^{0}-12\sqrt{5}c_{4}^{0}+24\sqrt{7}c_{6}^{0})].$$

$l_{{\text{C}}^{\prime }\text{P}1}$ determines the distance from the exit pupil center to the circle of least confusion in the vitreous humor. In other words, the circle of least confusion is set as the approximate focus point of the converging wavefront that passes through the exit pupil center. The distance of this focus point from the exit pupil center is derived from the mean curvature calculated based on differential geometry. The positions of these circles of least confusion located in the vitreous humor are set as the initial retinal points RP1. Then the rays emit from each point of RP1 are traced out of the eye model to obtain the SER for all field positions—denoted as MAP1.

If there were no aberrations in the eye, MAP1 would be same as MAP0. Due to the aberrations in ocular structure, a refinement of these points is in need to match the SERs of the eye model with MAP0.

• Step 2: Setting the retinal points with the same distance from RP1 (RP2)

**Fig. 3.**
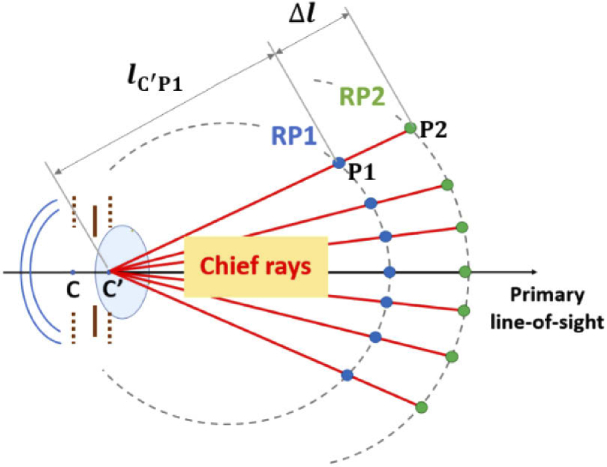
Locating the set of retinal points RP2 from RP1. The points C and C’ represent entrance and exit pupil, respectively, while P1 and P2 represent the pair of points each from RP1 and RP2 that situate along the same chief ray in the vitreous humor. The distance between P1 and P2 (Δl) is the same for all field positions.

To derive the slope $\Delta V_{out}/\Delta V_{in}$ for each visual field location, we need to obtain the SER map for another set of retinal points. As shown in Fig. 3, the retinal points RP2 are determined by translating all the points of RP1 along the chief rays in the vitreous humor by the same distance of Δl. For each visual field position, the distance from C’ to RP2 can be described by

(8) $$l_{{\text{C}}^{\prime }\text{P2}}=l_{{\text{C}}^{\prime }\text{P1}}+\Delta l.$$

As found in Section 3.1, the approximate linear relationship works for both axial and peripheral field angles. Thus, the value of Δl can be arbitrary, while a lower value may help increase the accuracy, because the refined retinal points are very close to RP1. In the examples presented in this study, Δl is set as 0.5 mm.

Similar to the method in Step 1, the SER map for the new set of retinal points RP2 is then calculated by ray tracing, which is denoted as MAP2.

• Step 3: Calculating the final retinal points RPf

Assume the SERs of the map MAP0, MAP1, MAP2 for a certain field position are represented by *M0*, *M1* and *M2*, respectively. The points of RPf, RP1, RP2 of the same field position are denoted as Pf, P1 and P2. Based on the linear relationship stated in Eq. (6), we get

(9) $$\frac{\Delta V_{out}}{\Delta V_{in}}=\frac{M0-M1}{\frac{n_{vi}}{l_{{\text{C}}^{\prime }\text{Pf}}}-\frac{n_{vi}}{l_{{\text{C}}^{\prime }\text{P}1}}}=\frac{M2-M1}{\frac{n_{vi}}{l_{{\text{C}}^{\prime }\text{P}2}}-\frac{n_{vi}}{l_{{\text{C}}^{\prime }\text{P}1}}}.$$

Combining Eq. (8) and (9), the distance from the exit pupil center to the final retinal point Pf can be determined by

(10) $$l_{{\text{C}}^{\prime }\text{Pf}}=\frac{M0-M1}{M2-M1}\cdot \frac{-\Delta l}{(l{}_{{\text{C}}^{\prime }\text{P}1}+\Delta l)l{}_{{\text{C}}^{\prime }\text{P}1}}+l{}_{{\text{C}}^{\prime }\text{P}1}.$$

Perform Eq. (10) for all the field positions involved in MAP0, the retinal contour composed of the final set of retinal points RPf can thus be located.

### 2.4 Optical analysis procedure

#### 2.4.1 Verification on the realistic eye model

The validity of the linear correlation theory and the performance of the proposed retinal modelling method are tested on a realistic eye model constructed from measurement data. The structure of the eye model is based on the adaptive model proposed by Navarro [20] with the crystalline lens replaced by the ‘physiology-like’ lens model [21] to achieve a better imitation of the lens structure of children. In this way, the toric and irregular shapes of the corneal surfaces, misalignment and tilts of ocular components, and the detailed gradient index distribution of crystalline lens are all incorporated so that the maximum anatomic similarity to the real eye structure can be achieved.

Parameters of the eye model are shown in Table 1. The ocular data—when available—are set based on measurement data on subjects of age 11 yr. Details of the applied measurement studies are shown in Table 2. The iris plane is assumed to be always tangential to the anterior crystalline lens surface, and the iris center coincides with the vertex point of anterior lens surface. Thus, the orientational parameters of the lens apply to the iris-and-lens combination with respect to the anterior lens vertex. The keratometric axis is set as the Z axis of the coordinate system for the eye model, so that all the intraocular distances are referenced to this axis. The primary line-of-sight is assumed to be parallel to the keratometric axis, which determines the corneal sighting center 0.083 mm to the temporal side and 0.03 mm to superior side of the vertex normal—within the range of measured data [22].

**Table 1.** Ocular parameters of the eye model

Ocular surface	Radius of curvature^*d*^ (mm)	Asphericity	Diameter *D* (mm)	Euler angles (α,β,γ)(°)	Decentration $\left(x_{0},y_{0}\right)$(mm)	Distance to the next surface	Refractive index^*a*^
Anterior corneal surface	(7.58,7.90)	(−0.3769, −0.3193)^*c*^	9	(0.574, −1.106, 60)	(−0.1544, −0.0697)	0.55	1.376
Posterior corneal surface	(6.2, 5.99)	(−0.56, −0.5636)^*c*^	9	(−2.251, −2.67,0)	(−0.361, −0.332)	3.068	1.335
Anterior lens surface	11.58	0.00046	8.574	(0, −3.7, 0)	(0.113, 0)	3.436	See below
Posterior lens surface	6.303	0.00025	8.574	N.A.	N.A.	N.A.	1.336
Other definitive parameters of the lens model^*b*^	Anterior versus total thickness ratio $T_{a}/T=0.45$;						
	Subtended angle of conic external surfaces $\theta_{a}=\theta_{p}=120^{\text{o}}$						
	*p* = 3.312, *q* = 1.598, $\left(n_{0},n_{s\_a},n_{s\_p},n_{s\_e})=(1.401,1.366,1.38,1.356\right)$, so that in the lens coordinate system (*z*=0 at lens equatorial center point): $\left\{ \begin{array}{l} \frac{z^{2}}{{\left(T_{a}\times \sqrt[{p}]{\frac{n_{s\_e}-n_{0}}{n_{s\_a}-n_{0}}}\times \gamma \right)}^{2}}+\frac{x^{2}+y^{2}}{{\left(D/2\right)}^{2}\gamma^{q+1}}=1,\;z\leq 0\\ \frac{z^{2}}{{\left(T_{p}\times \sqrt[{p}]{\frac{n_{s\_e}-n_{0}}{n_{s\_p}-n_{0}}}\times \gamma \right)}^{2}}+\frac{x^{2}+y^{2}}{{\left(D/2\right)}^{2}\gamma^{q+1}}=1,\;z\geq 0 \end{array}\right.,\text{ where }\gamma =\sqrt[{p}]{\frac{n-n_{0}}{n_{s\_e}-n_{0}}}.$						

^*a*^ All refractive indices are referenced at the wavelength of 589 nm.

^*b*^ The exact meanings of the lens parameters were given in the study by Li and Fang [21].

^*c*^ The Zernike coefficients (diameter 9 mm) of the anterior and posterior corneal surface are set as the mean values shown in Fig. 1 of the paper by Navarro et al. [26].

^*d*^ The radii of curvature for the anterior and posterior surface are self-defined within the range of measured data [27,28] to induce astigmatism of around 2 D.

**Table 2.** Summary of subjects’ information of the measurement studies applied for the eye model

Measurement studies	Sample size	Age	SER	Parameters applied in models
Mutti et al. (1998) [29]	994	6 ∼ 14 yr	−9.64 ∼ 4.62 D	Radii of curvature of lens external surfaces
Navarro et al. (2013) [26,30]	211	4 ∼ 79 yr	Not available	Anterior and posterior corneal surface topography
Ishii et al. (2013) [31]	25	0 ∼ 6 yr	0.24 +/−1.04 D	Lens diameter and external surface asphericity
Khan et al. (2018) [32]	38	18∼29 yr	−8.0 ∼ +1.0 D	Lens gradient index parameters
Rozema et al. (2019) [27]	1302	7 ∼ 13 yr	−5 ∼ 0.5 D	Intraocular distances and paraxial lens power^*a*^
Lan et al. (2019) [23]	82	10 ∼ 14 yr	0.03 +/- 0.28 D	Peripheral map of SER for emmetropic models
Lin et al. (2020) [24]	23	8 ∼ 17 yr	−5.36 ∼ −1.67 D	Peripheral map of SER for myopic models

^*a*^ Paraxial lens power is applied to determine the lens exponent parameter *q*. The details can be seen in Li and Fang [21].

**Fig. 4.**
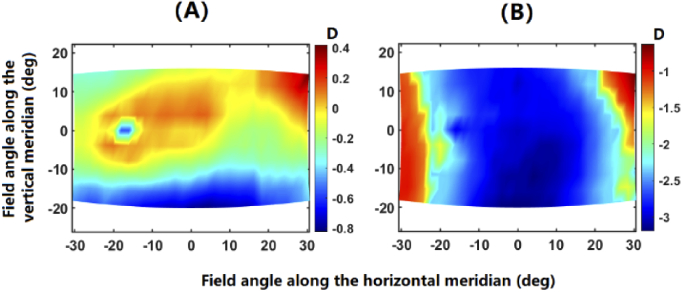
The input maps of SER (MAP0) for verifying the proposed method for retinal surface modelling, which is obtained by adding the central SERs of 0 D and −3 D to the relative peripheral maps of SER extracted from the measurement studies of Lan et al. [23] (A) and Lin et al. [24] (B) for emmetropes and myopes, respectively.

To evaluate the performance of the proposed retinal-locating method, three types of SER maps have been separately applied to construct the retinal contour: (1) planar map of 0D, (2) SER map for emmetropic eyes, and (3) SER map measured on myopic eyes. The emmetropic and myopic maps reported by the studies by Lan et al. [23] and Lin et al. [24], respectively, were applied in this study for the purpose of demonstration. The details of their measured subjects are shown in Table 2. However, the visual field specification of both studies is based on the Cartesian method. Their maps are transformed into the polar representation method by Eq. (1.a) and (1.b). The central SERs for the emmetropic and myopic maps are here set as 0 D and −3 D, respectively. The transformed SER maps are shown in Fig. 4, which were obtained by adding the central SERs to the relative peripheral SER maps (the original map of SER subtracted by the central SER) measured by Lan et al. and Lin et al., respectively. It should be noted that for all the field maps present in this paper, the positive values on the abscissa correspond to the temporal visual field along the horizontal meridian, while positive values on the ordinate associate with the inferior visual field along the vertical meridian.

In previous studies of eye models, the retinal contour is commonly represented by an ellipsoid or sphere. This simplification has the advantage of providing geometric features that are convenient for analysis, but it may not be capable of explaining some of the irregularities in the peripheral aberrations presented in the real human eye. To evaluate the capability in generalized ellipsoid model in reproducing peripheral SER distribution, the retinal points (RPf) obtained by the retinal-locating method are fit to a general ellipsoid, using the least squares method proposed by Navarro, et al. [25]. After obtaining the geometric parameters of the ellipsoid, the retinal points are calculated by finding the intersection points of the chief rays to the retinal contour. Then, the peripheral SER is calculated for those points and compared with the SER field map produced by RPf, namely MAPf.

#### 2.4.2 Implementation

Given the structural parameters of the eye model, the entrance pupil center can be found by tracing rays from iris center out of the cornea and find the position of the circle of least confusion for the emergent wavefront by Eq. (7). The exit pupil center can be determined in the same way by ray-tracing throughout the crystalline lens. The coordinate system (LoS system) for reporting the wavefront aberrations is determined based on ISO standard 24157: 2008. As shown in Fig. 1(A), the Z axis of the LoS system is the line-of-sight pointing toward the object space, while the X and Y axes are the projections of the X and Y axes in ocular system to the entrance pupil plane. Notice that the X axis of LoS system is opposite in direction to the X axis of ocular system. In this paper, Zernike coefficients up to the 6^th^ order are fit to all the wavefront aberrations to derive SER based on the formula

(11) $$SER=-\frac{1}{R^{2}}\cdot (4\sqrt{3}c_{2}^{0}-12\sqrt{5}c_{4}^{0}+24\sqrt{7}c_{6}^{0}),$$

where *R* is the diameter of the entrance pupil and is set as 4 mm for all the field angles.

Three-dimensional finite ray tracing is performed through the eye model using a set of custom-built MATLAB programs constructed based on Snell’s law in the vector form. The refracting functions are built separately for each type of surface geometry applied in the eye model [21]. Specifically, an iterative computing method [33] is applied to find the intersection points of the rays with the corneal surface which is a combination of biconicoid surface with Zernike polynomials. When tracing rays through a decentered or tilted refracting surface or component, transformations of the coordinates are performed for the ray parameters before applying the refraction equations. The crystalline lens model is set as 200 layers to approach the gradient index profiles. Therefore, more than 10,000 rays are traced to achieve high accuracy in wavefront extraction. Moreover, the boundary of the iris and emit rays are set large enough so that the minimum radius of the entrance pupil center at visual field of 40 deg is larger than 2 mm.

## 3. Results

### 3.1 Verification of the linear correlation theory

**Fig. 5.**
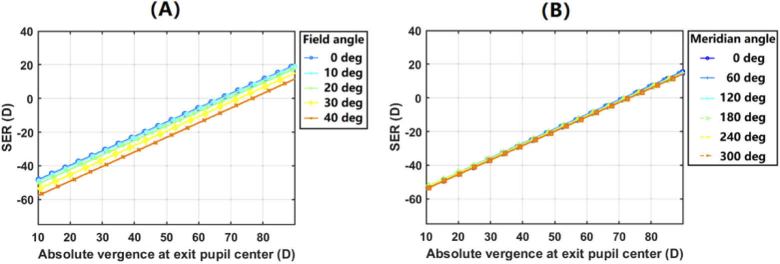
Change of spherical equivalent power (SER) with respect to the absolute vergence of wavefront at exit pupil center (A) over field angles of 0 to 40 deg along the 0 deg meridian, and (B) over 30 deg field angle along meridians from 0 to 300 deg.

The linear correlation relationship is tested over multiple visual fields on the realistic eye model (Table 1), by computing SER for a series of vergence values of the wavefront at exit pupil center for each field angle. The results over (1) field angle from 0 to 40 deg along 0 deg meridian and (2) 30 deg field angle along meridians from 0 to 300 deg are shown in Fig. 5(A) and (B), respectively. It can be observed that the linearity in the trendlines is evident in both axial and peripheral field angles.

**Fig. 6.**
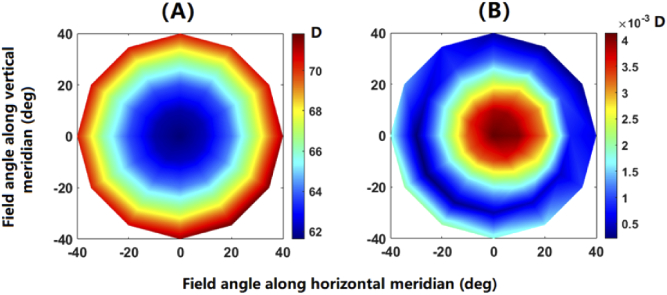
Field distribution of paraxial optical power (A), and root-mean-square error of the linear regression fit (B).

Linear regression fit was performed to each of the trendlines over field angles up to 40 deg (in steps of 10 deg) along the meridians from 0 to 360 deg (in steps of 30 deg). Based on Eq. (6), the distribution of paraxial optical power can thus be derived, as shown in Fig. 6(A). The paraxial power increases with eccentricity from around 62 D at central field to around 71 D at the field angle of 40 deg. Despite the irregularities in the ocular structures, the pattern of peripheral optical power is approximately rotationally symmetric. The root-mean-square of the linear fit is shown in Fig. 6(B). The maximum RMS error below 0.005 D indicates a high level of linearity in the trendlines for the investigated visual field.

### 3.2 Evaluation of the precision in peripheral defocus reproduction

**Fig. 7.**
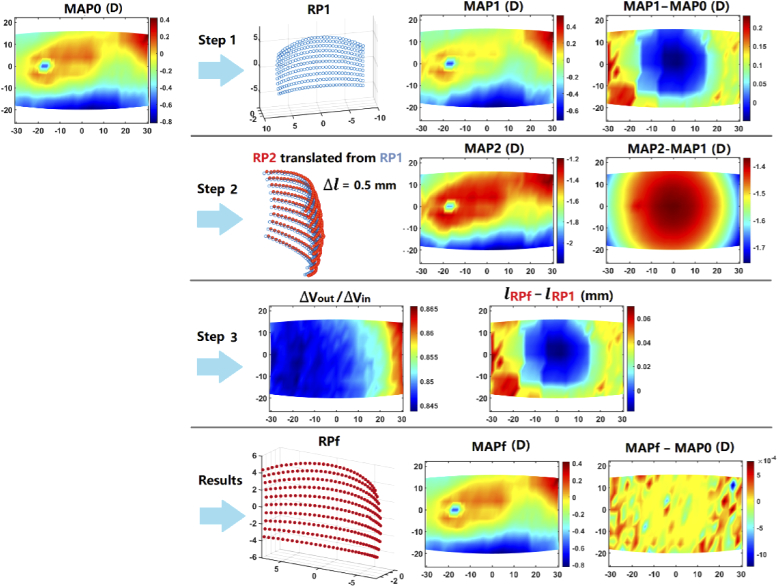
The procedure of obtaining retinal contour to reproduce the peripheral SER measured on emmetropic children. $l_{\text{RPf}}-l_{\text{RP1}}$ means the distance from RP1 to RPf along the chief rays.

Using the retinal-locating method proposed in Section 2.3, the retinal contours were derived for emmetropic, myopic and zero maps of SER, respectively. The details in the procedure for obtaining the retinal points for the emmetropic map is demonstrated in Fig. 7. The first set of retinal points RP1 are derived directly from the targeted map MAP0. MAP1 presents a close resemblance to the MAP0, but with a difference of 0.15 to 0.22 D at the peripheral angles. A new set of retinal points RP2 is then obtained by translating RP1 along the lines-of-sight by 0.5 mm, so that the SER map of RP2 (MAP2) can be obtained. By inputting MAP0, MAP1, MAP2, $l_{\text{RP1}}$ and Δl into Eq. (10), the final set of retinal points RPf can be determined. As shown in Fig. 7, step 3 helps refine the locations of RP1 by −0.02 to 0.07 mm. The result of verification shows that the difference between the SER maps of MAPf (the SER map of RPf) and the MAP0 are less than 0.001 D.

**Fig. 8.**
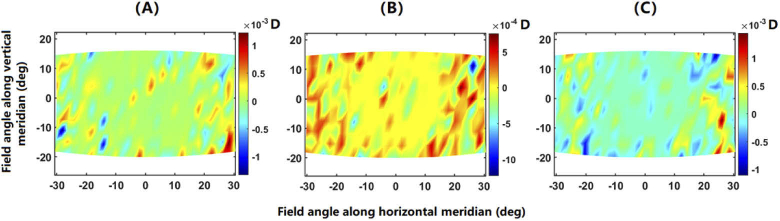
The error (MAPf – MAP0) of the constructed retinal contours in reproducing (A) the zero map, (B) the emmetropic map and (C) the myopic map, respectively.

The error (MAPf minus MAP0) of the obtained retinal contours in reproducing SER maps is shown in Fig. 8. As can be seen, the maximum error is within 0.002 D across the visual field, indicating the supreme performance of the proposed method in reproducing SER distribution.

### 3.3 Significance of exact retinal surface modelling

In this section, the retinal contours of the eye models for three types of SER maps are each fit to a general ellipsoid incorporating both decentration and orientation. The results are summarized in Table 3. Three principal radii of the ellipsoid are transformed into the biconic parameters ($R_{x},R_{y},Q_{x},Q_{y}$) at the apex, where the subscripts x and y represent the direction of two principal (maximum and minimum) curvatures that are closest to the X and Y axes of the eye system, respectively. The decentration of the apex of the ellipsoid with respect to the global eye system is represented by ($x_{0},y_{0}$), while the Euler angles (α,β,γ) represents the vertical tilt, horizontal tip and meridional rotation. By finding the intersection point of an axial ray with the fit ellipsoid, the axial length of the eye model (*AL*) can be obtained as the axial distance from the intersection point to anterior corneal surface.

**Table 3.** The geometric parameters of the ellipsoid fit to the retinal contours computed to reproduce the zero, emmetropic and myopic peripheral SER.

Map of SER	Zero	Emmetropic	Myopic
$R_{x}$(mm)	10.54	11.12	9.75
$R_{y}$(mm)	9.98	11.81	11.12
$Q_{x}$	−0.131	0.152	0.107
$Q_{y}$	−0.177	0.224	0.262
$x_{0}$(mm)	0.778	−3.688	−1.541
$y_{0}$(mm)	−0.875	−0.504	2.006
AL (mm)	23.69	23.68	24.80
$\alpha^{(}\circ )$	3.51	2.52	11.92
$\beta^{(}\circ )$	3.57	−20.65	−10.38
$\gamma^{(}\circ )$	34.26	6.33	1.20
RMS error of fit (mm)	0.0082	0.0346	0.1093

As shown in Table 3, the level of RMS fit error is the largest for myopic model (0.1093 mm), followed by emmetropic model (0.0346 mm) and the model with zero SER map (0.0082 mm), which indicates that the retinal shapes of myopic eyes deviate more from an ellipsoid than emmetropic eyes. For the retinal contours based on emmetropic and myopic SER maps, the horizontal radius of curvature is found lower than vertical radius of curvature, which is opposite in the model of zero SER map. Moreover, the vertex radii of retinal contours are found to be larger in the emmetropic model than myopic model, and the difference between horizontal and vertical radii is larger in the myopic model (1.37 mm) than emmetropic model (0.69 mm). Furthermore, oblate shape is found for both emmetropic and myopic retinas. Specifically, the oblateness near horizontal meridian for the myopic retina is lower than that of the emmetropic retina. These patterns are consistent with the SER maps as shown in Fig. 4, where the relative myopic defocus is presented near the vertical meridian of both maps, which requires a larger radius of curvature than that of the retinal contour for zero map. Along the horizontal meridian, however, emmetropic model remains relative myopic in peripheral field while a significant peripheral hyperopic defocus is present in the myopic model. This pattern agrees with the order of horizontal radii of three models: myopic (9.75 mm) < zero (10.54 mm) < emmetropic (11.12 mm).

Both ocular structure and the SER map play a significant role on the decentration and orientation of the retinal contour. The amount of rotation is found to be the largest for the horizontal tip in emmetropic model of −20.65 deg, which is almost twice the amount for myopic model (−10.38 deg). The negative values indicate that the ellipsoid centers are more nasal to the vertex point. For the vertical meridian, the ellipsoid center is found more superior to the vertex in all the retinal models. Meanwhile, a nasal decentration of the vertex is found in most eye models, while the vertical decentrations of emmetropic and myopic models show opposite signs.

**Fig. 9.**
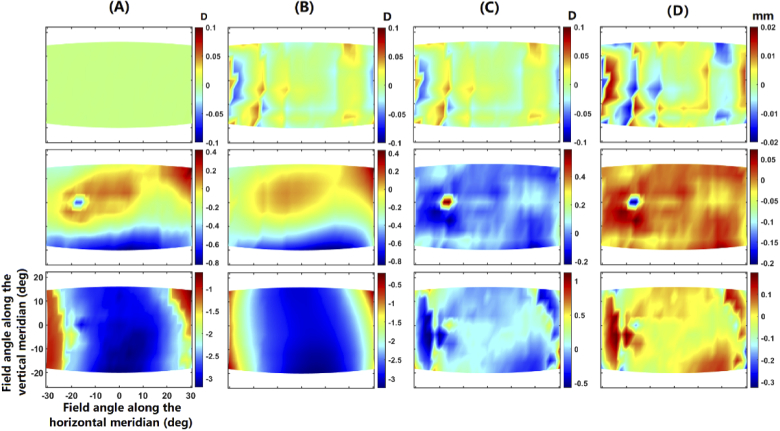
The evaluation of fitting the retinal points by a general ellipsoid: (A) SER maps for the original retinal points (MAPf); (B) SER maps for the ellipsoid retinal points (MAPe); (C) the deviation between the maps of MAPf and MAPe; (D) the distance from the ellipsoid to the original retinal points along lines-of-sight. The first, second and third rows are the results for the zero, emmetropic and myopic maps of SER, respectively.

To reveal the capability of ellipsoid retinal surface modelling in reproducing the unique patterns present in the peripheral SER maps, the peripheral SER maps are calculated from the retinal points on the ellipsoid over the same sampled field positions. The first, second and third rows of Fig. 9 show the results for the zero, emmetropic and myopic models, respectively. The four columns respectively present the SER map of the original retinal points (MAPf), SER map of the fit ellipsoid (MAPe), the error in SER map reproduction (MAPe subtracted by MAPf), and the distance from the fit ellipsoid to the original retinal points (RPf) along the chief rays. As can be seen, the ellipsoid model can reproduce a rough pattern in the field map of SER, whereas errors of 0.2∼0.5 D can occur at some field positions. These field positions with significant errors of reproduction are caused by a deviation of around 0.1∼0.2 mm. Moreover, the error for the ellipsoid model is the largest for the myopic map, followed by the emmetropic map and the zero map.

## 4. Discussion

**Fig. 10.**
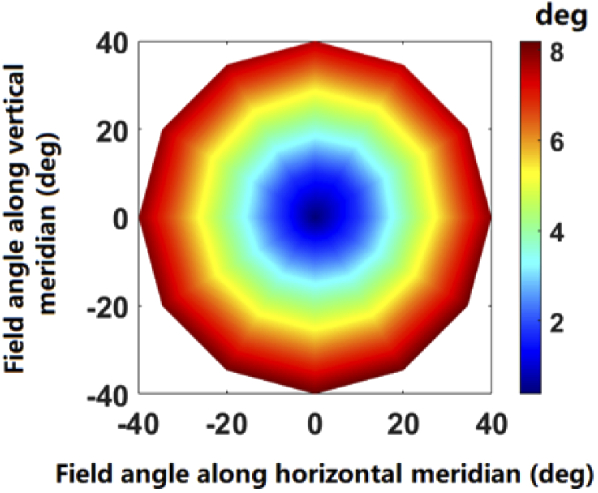
The angle between the section of chief rays in air (object space) and in vitreous humor

In the section 3.1, the linear correlation theory is proved to be effective across 80 deg diameter of the visual field for the realistic eye model. The theory is derived from paraxial optical theory, which is based on the assumption of paraxial imaging through rotationally symmetric optical system. Interestingly, the findings show that this paraxial optical behavior is also present in peripheral visual fields of the eye model. The phenomenon may be explained from two aspects. First, the vergence of the wavefront calculated based on Eq. (7) is essentially derived from the average curvature at the intersection point of the chief ray with the wavefront. Second, it has been found that the angle between the air-side and vitreous side of the chief ray is relatively small. As shown in Fig. 10, the maximum angle is around 8 deg at the most peripheral field of 40 deg. Therefore, the chief ray for each field angle can be approximated as close to the paraxial optical axis for each field. To our knowledge, this linear correlation phenomenon has not been confirmed in previous studies on human eye. Considering that the complex misaligned ocular structure does not strictly meet the conditions for paraxial optics, more rigorous theoretical explanations need to be developed in future studies.

Supported by this finding, we propose a computing method that can be used to reproduce the field map of SER by locating a set of retinal points on the retinal contour for a given optical structure of the eye model. To evaluate the performance of the proposed method, the retinal surface modelling procedure is performed separately to reproduce three types of SER field maps, respectively. Two of the field maps are extracted from the average relative peripheral SER measured on emmetropic and myopic children, which possess the natural irregularities in the pattern of distribution, while the zero map serves as a reference for retinal contour providing zero SER across the field. As shown by the results, the maximum error of reproducing the SER map is less than 0.002 D across all the cases. To our knowledge, this error for the peripheral SER is significantly lower than the level achieved by previous customized eye models. In a recent work by Liu and Thibos [10], customized eye models are constructed to reproduce the peripheral aberrations measured on the individual, where the reconstruction of SER can deviate from the measured value by 0.6 D at some field positions. In the wide-field emmetropic eye model proposed by Akram, et al. [9], the deviation of the modelled SER from the measured SER can reach around 0.25 D.

The retinal contour in the previous optical eye models was mainly represented by spherical, conic or biconic surfaces. However, it is likely that this simplification may not be able to reconstruct certain irregular features in the patterns of peripheral SER. In this study, a general ellipsoid is fit to the set of retinal points for the proposed eye model with three variations separately. It has been found that the fit of error is more significant in the myopic model compared with emmetropic model. Although a general pattern of the SER map can be achieved by the general ellipsoid modelling, the error of reproduction can reach 0.5 D for some field positions. Thus, to maximize the accuracy in reproducing the peripheral defocus, the retinal contour should be modelled as a set of points with third order interpolation, rather than conic or biconic surfaces. With the computing method proposed in this paper, the reproduction of SER can be achieved for any number of visual field points from measured data.

On the other hand, the retinal shapes obtained by the proposed method generally conform to similar patterns found by measurement data, as follows:

• Both myopic and emmetropic models have oblate retinas, while the oblateness is lower for the myopes [34–38].
• The radius of curvature of myopic retinas are smaller than emmetropic retinas [34,36].
• The vertex radius of curvature in the horizontal meridian is generally lower than that of the vertical meridian [34,36,39].

However, it needs to be mentioned that the retinal contour obtained by the proposed method is mainly aimed for reproducing the 2D map of SER, instead of obtaining the true contour of the retina. The accuracy of reproducing the retinal contour depends on the accurate modelling of the ocular components. Similarly, the retinal contour obtained by the other optical techniques (including both optical coherence tomography and refraction-based modelling) relies on a predefined structure of the eye model [40–42], which can induce errors due to the uncertainties in the gradient index distribution of the crystalline lens. Therefore, MRI is commonly considered as the most reliable method for measuring the retinal contour. However, this method suffers from low resolution, typically around 0.5 mm [35]. As found in our study, an interval of 0.2 mm in the distance from the retinal point to the exit pupil center can induce a change of 0.5 D in the SER. Thus, the resolution of MRI is not high enough for the reproduction of SER with higher accuracy. Moreover, MRI is quite expensive and requires lots of time and effort for measurement, which prevent it from being widely applied to a large population of children. Therefore, more work needs to be done to measure the retinal contour with high accuracy and reliability.

Up until now, the customized eye models were mostly constructed based on the optimization method, where certain unmeasured structural parameters of the human eye were optimized to achieve maximum similarity of the modelled optical features to the measured data. However, this method has significant limitations for reproducing more details present in the realistic human eye. With increasing number of variables for optimization, the cost of time could be unacceptable. A solution to this dilemma is to establish the relationship between the structural and optical properties of the human eye and use this relationship to directly derive certain ocular structures based on the optical features. Therefore, the peripheral-SER-based retinal modelling method proposed in this study is a great step in the customized eye modelling by significantly improving both reproduction accuracy and calculation efficiency to the next level. Moreover, this method can also be applied in building generic eye models for the myopic eyes with the focus on reproducing the average peripheral SER distribution found in the population.

Peripheral defocus is commonly believed to play a significant role in the development and progression of myopia. However, it is still unclear what amount of defocus is significant enough to make a difference, and what extent of the visual field can produce a significant effect. Therefore, it is critical for eye models to be capable of achieving the full distribution of SER measured in the individual eye. On the other hand, the quality of the image received by the retinal is dominated by defocus, followed by astigmatism and then higher order aberrations. Therefore, the accurate reproduction of SER should be prioritized compared with other optical features in the human eye modelling for evaluating optical methods for myopia control. Specifically, the eye model with customized peripheral defocus distribution is fundamental for the design and evaluation of customized optical corrections.

However, the myopic/hyperopic defocus signal sensed by the retina depends on the optical blur which is contributed by both refractions and higher order aberrations. Due to astigmatism, coma, and other non-symmetric aberrations, the optical blur is generally anisotropic at peripheral angles, which is very likely a clue to the direction of the defocus. The ultimate goal of wide-angle eye modelling is to reproduce both refractions and higher order aberrations of the human eye for the total visual field, which cannot be achieved efficiently by the conventional optimization methods. The reproduction of astigmatism and higher-order aberrations relies on the establishment of a direct relationship between the modelling of ocular components and the distribution of peripheral aberrations, which will be investigated in our future studies.

## 5. Conclusions

In this study, a linear relationship has been found between the spherical equivalent refraction and vergence of the wavefront at exit pupil center. The linear relationship has been verified to work over both axial and peripheral visual field of the realistic eye model. Based on that finding, a retinal-locating method is proposed that can reproduce the peripheral defocus map with high resolution and accuracy. The proposed scheme has been verified for both emmetropic and myopic maps of peripheral SER measured on human eye. The maximum error in reproducing the field map of SER is achieved less than 0.002 D for the first time. Furthermore, the capability of ellipsoid retinal shape modelling in reproducing natural pattern of SER distribution is tested. The significant error of fit found in the myopic model proves the fact that ellipsoid description is not sufficient to faithfully reproduce the defocus across the field of view.

In summary, this work is a fundamental step for customized human eye modelling by significantly improving the precision and efficiency to the next level. Without using optimization procedure, the points of the retinal contour can be derived directly from the field map of peripheral defocus. The proposed method is of great value for the design and evaluation for myopia control that are dedicated for individual correction.

A fuller reproduction of astigmatism and the higher aberrations depends also on the realistic modelling of the ocular components. The accuracy of the modeled retinal contour with respect to the real one depends on the precision of modelling the ocular components. However, it is still unclear whether this can be achieved with the current measurement technology. In the future study, the proposed method will be tested and further developed by building customized eye models from the individual measurement data.

## Data Availability

Data underlying the results presented in this paper are not publicly available at this time but may be obtained from the authors upon reasonable request.
